# Supplementary material for: WordCommentsAnalyzer: A windows software tool for qualitative research
Source: F1000Res. 2018 Sep 4;7:536. Originally published 2018 May 3. [Version 2] doi: 10.12688/f1000research.14819.2 (PMC6137411; doi:10.12688/f1000research.14819.2)
Supplement: Supplementary file 3 [file f1000research-7-17614-s0002.tgz › b2a79496-4ada-47c4-9682-a99ce867836d.zip!/Eight Iranian Health Journals/Yafteh _20.docx]

© Yafteh  . This work is licensed under CC BY 4.0 (https://creativecommons.org/licenses/by/4.0/).

Volume & Issue: دوره 15 - شماره 3

Title: مقایسه آنالیز ژنوتیپی KIR/HLA در جمعیت‌های لر و ایرانی

http://yafte.lums.ac.ir/article-1-1319-fa.html

مقدمه: پذیرنده‌های شبه ایمونوگلبولینی سلول کشنده (KIR) یک خانواده از پذیرنده‌های مهار کنندگی و فعال کنندگی هستند که به طور عمده توسط سلول‌های کشنده طبیعی NK)) بیان می‌شوند. پروتئین‌های KIR به عنوان پذیرنده‌هایی عمل می‌کنند که مولکول‌های آنتی‌ژن لکوسیتی انسان (HLA) کلاس I را شناسایی می‌کنند. KIRها و لیگاندهای HLA کلاس I آنها در پاتوژنز انواع مختلف بیماری‌ها مشارکت دارند. هدف این مطالعه آنالیز ژنوتیپی KIR/HLA در قوم لر برای اولین بار بود. مواد و روش‌ها: ‌در این مطالعه 100 فرد سالم غیرخویشاوند لر از نظر ژن‌های KIR و لیگاندهای HLA به روش واکنش زنجیره‌ای پلیمراز با استفاده از پرایمرهای اختصاصی توالی تایپ گردیدند. در نهایت، فراوانی ژن‌ها و ژنوتیپ‌های KIR و لیگاندهای HLA و ترکیبات KIR-HLA در جمعیت لر با جمعیت ایرانی مقایسه شد. یافته‌ها: در جمعیت لر 22 ژنوتیپ KIR پیدا شدند و تمام ژن‌های KIR نیز مشاهده گردیدند. شایع‌ترین ژن‌های غیر چارچوبی در جمعیت لر KIR2DL1 و KIR2DP1 با 98٪ و KIR3DL1 و KIR2DS4 با 96٪ بودند. شایع ترین ژنوتیپ KIR مشاهده شده در جمعیت لر، ژنوتیپ AA با فراوانی 29٪ بود. از میان ژنوتیپ‌های لیگاند HLA، ژنوتیپ شماره 1 با فراوانی 35٪ بیشترین فراوانی را در جمعیت لر داشت. شایع‌ترین ترکیبات KIR-HLA مهاری و فعال کنندگی در جمعیت لر به ترتیب KIR2DL2/3+HLA-C1 با فراوانی 75٪ و KIR2DS2+HLA-C1 با فراوانی 47٪ بودند. بحث و نتیجه‌گیری: نتایج نشان می دهد که فراوانی ژن‌ها و ژنوتیپ‌های KIR و لیگاندهای HLA و ترکیبات KIR-HLA در جمعیت لر دارای ویژگی‌های کلی گزارش شده در جمعیت‌ ایرانی می‌باشد، ولی با کاهش یا افزایش برخی فراوانی‌های هنوز هم منحصر به فرد است.

Title: تاثیر تمرینات هوازی و بی‌هوازی براضطراب و میزان ترشح هورمون کورتیزول خون

http://yafte.lums.ac.ir/article-1-1320-fa.html

مقدمه: هدف این مطالعه، بررسی تاثیر تمرینات هوازی و بی‌هوازی بر اضطراب و میزان ترشح هورمون کورتیزول خون کشتی‌گیران جوان بود. مواد و روش‌ها: در این تحقیق نیمه‌تجربی، از بین 200 کشتی‌گیر، 20 نفر (سن؛17-20 سال) داوطلب بر اساس دارا بودن درجات بالایی اضطراب انتخاب و به صورت تصادفی به دو گروه 10 نفری تمرین هوازی و بی‌هوازی تقسیم شدند. آزمون‌های استفاده شده در این مطالعه شامل دویدن مسافت 2400 متر برای گروه هوازی و دویدن مسافت 400 متری در 3 نوبت برای گروه بی‌هوازی بود. یافته‌ها: میانگین نمرات اضطراب گروه هوازی بلافاصله پس از آزمون کاهش یافت، در حالیکه میانگین نمرات اضطراب گروه بی‌هوازی بلافاصله پس از آزمون افزایش پیدا کرد(015/0>P). بلافاصله پس از آزمون یک جلسه تمرین بی‌هوازی، میانگین غلظت هورمون کورتیزول افزایش پیدا کرد، درحالی که میانگین غلظت هورمون کورتیزول درگروه تمرین هوازی کاهش پیدا کرد(01/0 >P). بحث و نتیجه‌گیری: با توجه به نتایج بدست آمده از تحقیق حاضر می‌توان عنوان نمود که یک جلسه تمرین هوازی در کاهش اضطراب کشتی‌گیران مؤثر بوده است و همچنین تداوم این تمرینات در دراز مدت با کاهش سطوح هورمون کورتیزول موجب کاهش اضطراب و ایجاد شرایط روحی و روانی متعادل در ورزشکاران می‌گردد.

Title: بررسی اپیدمیولوژیک سرطانهای بخش فوقانی دستگاه گوارش در بیماران مراجعه کننده به بیمارستان شهدای عشایر خرم آباد

http://yafte.lums.ac.ir/article-1-1362-fa.html

مقدمه: با پیشرفت علوم پزشکی و کنترل بیماری‌های عفونی، سرطان‌ها در جوامع بشری بیش از پیش جلب توجه می-کنند.سرطان‌های بخش فوقانی دستگاه گوارش یکی از مهمترین سرطان‌ها بوده که شیوع بالایی در بسیاری از کشورها دارد. مواد و روش‌ها: یک بررسی کلی بر 73 بیمار مراجعه کننده با دیفساژی، درد اپی‌گاستر و کاهش وزن در مدت کوتاه که نتیجه بررسی پاتولوژیک آنها موید وجود بدخیمی در بخش فوقانی دستگاه گوارش بود از 15 فروردین 84 تا 15 فروردین 85 انجام شد. متغیرها در این مطالعه شامل سن و جنس، مصرف سیگار، سطح سواد، سابقه فامیلی سرطان، مصرف غذای چرب، مصرف چای داغ ، نوع پاتولوژیک سرطان و محل آناتومیک تومور بود.یافته‌ها: شیوع سرطان‌های بخش‌های فوقانی دستگاه گوارش در مردان دو برابر زنان بود. بیشترین شیوع سرطان های بخش‌های فوقانی دستگاه گوارش در دهه هفتم و سپس دهه هشتم زندگی بود. در این مطالعه بیشتر بیماران مصرف چای داغ را ذکر می‌کردند اما مصرف غذای چرب را ذکر نمی‌کردند. بحث و نتیجه‌گیری: سرطان مری و معده در استان لرستان و همچنین ایران از شیوع بالایی برخوردار می باشد که حدس زده می شود نقش عوامل تغذیه ای در ایجاد سرطان‌های فوق از مصرف سیگار مهم‌تر باشد.

Title: مقایسه پیش آگهی اعمال جراحی سینوس پیلونیدال در بیماران مراجعه کننده به بیمارستان شهدای عشایر خرم آباد در سالهای 1391-1383

http://yafte.lums.ac.ir/article-1-1363-fa.html

مقدمه: بیماری پیلونیدال شیوع رو به افزایشی دارد ( 26نفر از هر 100000) و عمدتا محدوده سنی 40-18سالگی را درگیر می کند. با توجه به اینکه محدوده سنی مذکور بهینه ترین زمان فعالیت اجتماعی و اقتصادی افراد می باشد، برآن شدیم که به ارزیابی پیش آگهی بیماران تحت جراحی سینوس پیلونیدال بیمارستان شهدای عشایر خرم آباد در سالهای 1391-1383 بپردازیم. مواد و روش‌ها: در قالب مطالعه همگروهی تاریخی ابتدا با مراجعه به واحد بایگانی بیمارستان شهدای عشایر خرم آباد و دریافت پرونده و شماره تماس بیماران مذکور تحت اعمال جراحی سینوس پیلونیدال (به سه روش مطرح) ، متغیرهایی چون سن، جنس، شغل، سابقه ابتلا به بیماریهای مشابه، روش جراحی به کار رفته، عوارض پس از درمان صورت گرفته و ... مورد بررسی و تحلیل آماری قرار خواهند گرفت . بحث و نتیجه‌گیری: در پایان مطالعه گزارش گردید که بیشترین عوارض پس از درمان سینوس پیلونیدال ،مربوط به روش جراحی باز بوده است. منجر به فاصله گرفتن بیش از حد بیمار از فعالیت های اجتماعی و اقتصادی نیز می گردد. یافته‌ها: تمامی بیماران تحت درمان با روش بسته با درن و یا بخیه در مقایسه با روش باز( حدود 50 درصد) رضایت بالا را مطرح کردند(04/0=P در بیماران مونث و002/0=P در بیماران مذکر). همچنین شایعترین عارضه مطرح در روشهای درمانی بسته، خونریزی از موضع بود. در حالی که در جراحی باز عمده بیماران از درد طولانی مدت موضع و در ادامه به ترتیب از عفونت موضع، خونریزی و عود سینوس پیلونیدال شاکی بوند(024/0=P در بیماران مونث و000/0=P در بیماران مذکر).

Title: اثر ورزش طولانی مدت بر میزان فعالیت آنزیم های آنتی اکسیدان بعد از بستن رگ کاروتید دررت

http://yafte.lums.ac.ir/article-1-1364-fa.html

مقدمه: مطالعات جدید نشان داده اند که ورزش منظم حساسیت مغز به آسیب های مغزی تحت شرایط پاتولوژیک مختلف را کاهش می دهد. هدف از مطالعه حاضر بررسی کردن نقش حفاظتی ورزش منظم بر هیپوکامپ بعد از هیپوپرفیوژن مغزی می باشد. مواد و روش‌ها: رتهایی از نژاد ویستار به صورت تصادفی به 4 گروه کنترل، هیپوپرفیوژن (دوم)، ورزش (سوم) و ورزش – هیپوپرفیوژن (چهارم) تقسیم شدند. حیوانات گروه های سوم و چهارم وادار شدند 2.5 ماه هر روز به مدت 1 ساعت برروی تردمیل با سرعت 17 متر در دقیقه بدوند. شریان کاروتید مشترک راست در حیوانات گروه چهارم، پس از پایان دوره ورزش با نخ بخیه بسته شد. در گروه دوم تنها عمل بستن رگ کاروتید صورت گرفت و گروه کنترل هیچکدام از اعمال فوق ( ورزش و بستن رگ کارتید) بر حیوانات انجام نگرفت. سپس 72 ساعت بعد از بستن کاروتید حیوانات کشته شدند و فعالیتهای آنزیم های آنتی اکسیدان (آنتی اکسیدان توتال، کاتالاز و سوپراکسید دیسموتاز) در هیپوکامپشان توسط کیت های الیزا اندازه گرفته شد. بحث و نتیجه‌گیری: این مطالعه نشان داد که ورزش اثر حفاظتی بر هیپوکامپ حیوانات از طریق افزایش فعالیت آنزیمهای آنتی اکسیدان بویژه سوپر اکسید دیسموتاز در 72 ساعت بعدازایجاد هیپوپرفیوژن دارد.به نظر می رسد مطالعه بیشتر در این زمینه برای دانستن مکانیسم ورزش مورد نیازمی‌ باشد. یافته‌ها: ورزش فعالیت کل آنزیم های آنتی اکسیدان را در هیپوکامپ چپ حیوانات گروه چهارم در مقایسه با گروه های دوم وکنترل به طور معناداری افزایش داد.در گروه دوم فعالیت آنزیم سوپراکسید دیسموتاز در هیپوکامپ کاهش یافت(05/0< p)، اما ورزش موفق گردید این مقدار را به مقدار گروه کنترل برساند. فعالیت آنزیم کاتالاز در گروه چهارم نسبت به گروه اول و دوم به طور معنا داری در اثرورزش افزایش یافت. فعالیت آنزیم های سوپراکسید دیسموتاز و کاتالاز بین نیمکره ها در هرگروه تفاوت معناداری را نشان نداد.

Title: تأثیر دراز مدت بستن دو طرفی لوله های رحمی بر تخمدان و رحم در موش بزرگ (مطالعه هیستوپاتولوژیک)

http://yafte.lums.ac.ir/article-1-1365-fa.html

مقدمه: هدف مطالعه بررسی تعداد انواع فولیکول های تخمدانی و تغییرات رحمی در 3 ، 6 و 9 ماه بعد از بستن دوطرفی لوله های رحمی در موش بزرگ بود. مواد و روش‌ها: در این مطالعه تجربی 60 موش بزرگ (اسپراگو-داولی) ماده دوماهه به دو گروه کنترل و گروه بستن لوله های رحمی (هر گروه 30 حیوان) تقسیم شدند. بستن لوله ها دوطرفی و شبیه روش پومروی انجام گرفت. هر سه ماه 10 حیوان از گروه کنترل و 10 حیوان از گروه بستن لوله ها به طور تصادفی انتخاب و سپس تخمدان و رحم چپ حیوان نمونه گیری و در محلول فرمال سالین ثابت گردید. برش های پارافینی (5 میکرون ضخامت) تهیه و به روش هماتوکسیلین-ائوزین و تریکروم رنگ آمیزی شدند. تغییرات آندومتر به طریقه کیفی و انواع فولیکول های تخمدانی شمارش گردیدند. داده ها با آزمون من – ویتنی با نرم افزار SPSS13 ارزیابی گردیدند(05/0< p). بحث و نتیجه‌گیری: بستن دو طرفی لوله های رحمی در موش بزرگ موجب تغییرات معنادار در تعداد انواع فولیکول های تحمدانی و جسم زرد نمی شود ولی ممکن است موجب القای التهاب آندومتر و هیپرپلازی کیستیک آندومتر در دراز مدت گردد. یافته‌ها: بین گروه کنترل و گروه بستن لوله ها بعد از 3 ،6 و 9 ماه تغییرات معناداری در تعداد انواع فولیکول های تخمدانی و جسم زرد وجود نداشت. ارتشاح متوسط تا شدید سلول های التهابی در آندومتر گروه بستن لوله ها مشاهده شد. برای اولین بار هیپرپلازی کیستیک آندومتر در20% حیوانات 9 ماه بعد از بستن لوله ها مشخص گردید.

Title: تأثیر آموزش روانی خانواده و درمان استاندارد پزشکی در سیر بهبودی و پیشگیری از عود علائم بیماران افسرده اساسی

http://yafte.lums.ac.ir/article-1-1366-fa.html

مقدمه: افسردگی یکی از شایع‌ترین اختلالات روانی عصر ماست. هدف از پ‍‍‍‍ژوهش حاضر تأثیر آموزش روانی خانواده در کنار درمان استاندارد پزشکی در سیر بهبودی و پیشگیری از عود علائم بیماران افسرده اساسی بود. بحث و نتیجه‌گیری: با درمان استاندارد پزشکی توأم با آموزش روانی خانواده در بیماران با اختلال افسردگی اساسی در مقایسه با درمان استاندارد پزشکی می‌تواند اثر بخشی بیشتری در بهبود و پیشگیری از عود علائم افسردگی داشته باشد. یافته‌ها: یافته‌های پژوهش ما نشان داد اختلاف معنی داری بین گروه تجربی و گروه کنترل در مرحله پس از مداخلات (001/0=p) و مرحله پیگیری (008/0=p) بدست آمد. مواد و روش‌ها: نوع مطالعه کار آزمایی بالینی بود. با استفاده از روش نمونه گیری در دسترس تعداد 60 نفر بیمار مبتلا به اختلال افسردگی اساسی به صورت تصادفی انتخاب و به دو گروه مساوی تقسیم شدند. دو گروه تحت درمان استاندارد پزشکی بوده، که خانواده یکی از این گروه‌ها 8 جلسه تحت آموزش روانی خانواده قرار گرفتند. ابزار مورد استفاده آزمون افسردگی بک (ІІBDI -) بود که در سه مرحله پیش از مداخله، پس از مداخله و 6 ماه پس از آخرین مداخلات (پیگیری) روی بیماران اجرا گردید. داده‌های پژوهشی از طریق تحلیل کوواریانس (آنکوا) مورد تجزیه و تحلیل قرار گرفت.

Title: بررسی وضعیت سلامت روانی پرستاران شاغل در بیمارستان‌های دولتی شهر خرم آباد

http://yafte.lums.ac.ir/article-1-1367-fa.html

مقدمه: پرستاران به دلیل مشکلات شغلی و شرایط سخت کاری بیشترین استرس شغلی را تجربه می‌کنند و در معرض آسیب بیشتری قرار دارند. پرستارانی که از سلامت عمومی خوبی برخوردار نیستند، قادر نخواهند بود که مراقبت‌های مؤثری از بیماران انجام دهند. با توجه به اهمیت ارتقاء سلامت روان پرستاران و نقش کلیدی آنها بر کیفیت ارائه خدمات پرستاری، محقق بر آن شد تا تحقیقی را در خصوص تعیین وضعیت سلامت روان در پرستاران انجام دهد. بحث و نتیجه‌گیری: با توجه به یافته مهم این پژوهش مبنی بر تأثیر مثبت و معنادار فعالیت ورزشی در سلامت روان، ضروری به نظر می‌رسد که پرستاران فعالیت‌های ورزشی منظم را در برنامه زندگی خود قرار دهند و بدین وسیله موجبات ارتقاء سلامت عمومی خود را فراهم آورند. یافته‌ها: در کل 78 نفر (2/46 درصد) سلامت روان مطلوب و 91 نفر (8/53 درصد) دارای اختلال در سلامت روان بودند. میزان سلامت روان کلی با میانگین 9/27 درصد و انحراف معیار 1/13 بدست آمد. در خصوص ابعاد سلامت عمومی بیشترین نشانه عدم سلامت روان در بعد عملکرد اجتماعی (5/9%) بود و کمترین نشانه عدم سلامت در بعد افسردگی (3%) بود. در بررسی ارتباط بین سلامت روان و ویژگی‌های دموگرافیک ( نوبت کاری، سن، جنس و وضعیت تأهل و سابقه کار و سطح درآمد) هیچگونه ارتباط معناداری مشاهده نگردید. اما بین فعالیت ورزشی با سلامت روان ارتباط معناداری مشاهده گردید. مواد و روش‌ها: پژوهش حاضر یک مطالعه توصیفی- مقطعی از نوع همبستگی می‌باشد که در کل 169 نفر از پرستاران شاغل در بیمارستان-های وابسته به دانشگاه علوم پزشکی لرستان مورد بررسی قرار گرفتند. گردآوری داده‌ها توسط دو پرسشنامه که شامل اطلاعات دموگرافیک و پرسشنامه سلامت روان (GHQ-28 ) بود انجام گرفت و اطلاعات جمع آوری شده با استفاده از روش‌های آماری توصیفی (میانگین و انحراف معیار) و آمار استنباطی (آزمون‌های chi-squar ، T- test و ANOVA) و با استفاده از نرم افزار spss مورد تجزیه و تحلیل قرار گرفت

Title: اندازه گیری ظرفیت آنتی اکسیدانی تام و فعالیت آنزیم های کاتالاز و سوپراکسید دیسموتاز سرم افراد سیگاری در مقایسه با افراد غیر سیگاری

http://yafte.lums.ac.ir/article-1-1368-fa.html

بحث و نتیجه‌گیری: نتایج بدست آمده نشان داد که استرس اکسیداتیو ناشی از کشیدن سیگار باعث کاهش سیستم دفاعی آنتی اکسیدانی بدن گردیده که در طولانی مدت می‌تواند باعث بروز بیماری های قلبی و عروقی در افراد سیگاری شود. مواد و روش‌ها: مطالعه بر روی 30 فرد داوطلب سیگاری (سن35- 25 سال) که روزانه بیش از 10 نخ سیگار مصرف می‌کردند، انجام گرفت. گروه شاهد نیز 30 فرد داوطلب غیر سیگاری (سن35-25 سال) بودند. نمونه‌های خون ناشتا از این افراد گرفته شد. میزان فعالیت سرمی آنزیم های کاتالاز، سوپراکسید دیسموتاز و ظرفیت آنتی اکسیدانی تام آنها سنجیده شد. داده‌ها به صورت میانگین±انحراف استاندارد توصیف شد و میانگین داده‌ها در دو گروه با آزمون T مستقل ارزیابی شد. برای آنالیز آماری نیز نرم افزار SPSS استفاده شد. یافته‌ها: ظرفیت تام آنتی اکسیدانی و فعالیت آنزیم های کاتالاز و سوپراکسید دیسموتاز در گروه آزمون کاهش آماری معناداری با گروه کنترل داشتند (05/0>p). مقدمه: استرس اکسیداتیو نقش مهمی را در پاتوژنز بسیاری از بیماری ها مانند سرطان ریه، بیماری های مزمن انسداد ریوی و آترواسکلروز دارد. دود سیگار باعث افزایش استرس اکسیداتیو از طریق تولید رادیکال‌های فعال اکسیژن و کاهش سیستم دفاع آنتی اکسیدانی بدن می‌شود. هدف از این مطالعه بررسی اثر دود سیگار در تغییر ظرفیت آنتی اکسیدانی تام و فعالیت آنزیم های کاتالاز و سوپراکسید دیسموتاز در افراد سیگاری است.

Title: مقایسه‌ سبک‌های دفاعی در بیماران مبتلا به سرطان سینه با گروه غیربالینی

http://yafte.lums.ac.ir/article-1-1369-fa.html

مقدمه: مکانیسم‌های دفاعی، مسئولیت محافظت از (من) در برابر ناملایمات، اضطراب‌ها و کاستی‌های محیطی و درونی را بر عهده دارند و تحت تأثیر سازه‌های مختلف شخصیتی ممکن است کنش‌وری بهنجار یا نابهنجار داشته باشند. پژوهش حاضر با هدف بررسی مکانیسم های دفاعی بیماران مبتلا به سرطان سینه و گروه غیربالینی انجام گرفت. بحث و نتیجه‌گیری: این پژوهش – چه از لحاظ گستردگی و چه از لحاظ موضوع – از معدودترین پژوهش‌هایی بود که در آن برخی از عوامل روانی در مبتلایان به سرطان سینه بررسی و همسو با نظریات قبلی مبنی بر ارتباط تنگاتنگ مؤلفه‌های روانی و بیماری‌های جسمانی، این نتیجه دست آمد که مبتلایان به سرطان سینه از مکانیسم‌هایی استفاده می‌کنند که نظریه‌پردازان روان‌پویشی آنها را دریچه‌ ای به سوی نابهنجاری جسمی و روانی می دانند. یافته‌ها: نتایج نشان داد که میانگین مکانیزم‌های دفاعی نابالغ و نوروتیک در گروه مبتلا به سرطان سینه به‌طور معناداری بیشتر از جامعه غیربالینی است و در مقابل میانگین مکانیزم‌های دفاعی بالغ در جامعه غیربالینی بیشتر از افراد مبتلا به سرطان‌سینه بوده است. مواد و روش‌ها: در این پژوهش 246 نفر از مبتلایان به سرطان سینه و 204 نفر از دانشجویان به عنوان نمومه پژوهش انتخاب شدند. ابزار گردآوری داده ها در این پژوهش پرسشنامه DSQ بود.

Title: ارزیابی سیتوتوکسیک نوعی دود دارویی روی دو رده سلول سرطانی دهانه رحم و حنجره (Hela و Kb) و یک رده نرمال (L929) به روش MTT

http://yafte.lums.ac.ir/article-1-1370-fa.html

مقدمه: دود طبی حاصله از منابع طبیعی سلامتی بخش در بسیاری از کشورهای دنیا استفاده می‌گردد. یکی از این دودهای دارویی، دود ناشی از سوختن سرگین الاغ ماده با نام «عنبر نسارا» است. عنبر نسارا در طب سنتی و عامیانه ایران برای درمان بیماری‌های عفونی باکتریایی و ویروسی، تومورها و کیست‌های واژینال کاربرد دارد. بحث و نتیجه‌گیری: دود دارویی عنبر نسارا، حاوی ترکیبات فرار متعددی است که آثار سیتوتوکسیک مشاهده شده را می‌توان به آنها نسبت داد. مطالعات بیشتر روی این نمونه‌ها ترکیبات مؤثره آن را مشخص خواهد نمود. یافته‌ها: نتایج نشان می‌دهد دود ناشی از سوختن عنبر نسارا روی رده‌های سلولی فوق‌الذکر در غلظت های معادل 2/0 میلی گرم یا کمتر اثر کشنده دارد. مواد و روش‌ها: در این مطالعه، سرگین الاغ ماده از شهرضا در استان اصفهان طی تابستان 1390 جمع‌آوری و توسط دستگاه طراحی شده، سوزانده شد. دود حاصله در لوله‌ای حاوی آب و n-هگزان که توسط آب و یخ احاطه شده بود، جمع آوری گردید. حلال در مجاورت هوا تبخیر و از نمونه خشک شده برای انجام آزمون سمیت سلولی به روش MTT، غلظت های 4/0، 3/0، 25/0، 2/0، 125/0، 1/0، 625% و 325% میلی‌گرم در میلی‌لیتر محیط کشت حاوی 1%، DMSO تهیه شد. اثر سمیت سلولی این غلظت ها پس از 48 ساعت انکوباسیون روی دو رده سلولی سرطانی (KB و Hela) و یک رده سلولی نرمال L929 از طریق تعیین درصد سلول های زنده بررسی گردید.

Title: اثر ماست پروبیوتیک بر پروتئین واکنشی C سرم و قند خون در بیماران دیابتی نوع دو

http://yafte.lums.ac.ir/article-1-1371-fa.html

مقدمه: التهاب در پیشرفت دیابت و بیماری‌های قلبی- عروقی ناشی از آن نقش مهمی ایفا می‌کند. مطالعه‌ حاضر با هدف بررسی اثرات ماست پروبیوتیک بر قند خون و عامل التهابی پروتئین واکنشی C در بیماران دیابتی نوع دو انجام گرفت. بحث و نتیجه‌گیری: مصرف ماست پروبیوتیک به عنوان درمان کمکی به بیماران دیابتی نوع دو قابل توصیه است. یافته‌ها: دریافت ماست پروبیوتیک منجر به کاهش معنی‌دار غلظت سرمی پروتئین واکنشی C شد (05/0 > P). غلظت قند خون ناشتا و هموگلوبین گلیکوزیله در گروه ماست پروبیو مصرف ماست پروبیوتیک به عنوان درمان کمکی به بیماران دیابتی نوع دو قابل توصیه است. تیک در مقایسه با گروه شاهد بعد از مداخله به طور معنی‌داری پایین‌تر بود (05/0 > P). مواد و روش‌ها: این کارآزمایی بالینی تصادفی دوسو کور روی 60 بیمار دیابتی نوع دو (23 مرد و 37 زن) در بیمارستان سینای تبریز انجام گرفت. افراد گروه آزمون، روزانه 300 گرم ماست پروبیوتیک حاوی لاکتوباسیلوس اسیدوفیلوس LA-5 و بیفیدوباکتریوم لاکتیس BB-12 و افراد گروه شاهد، روزانه 300 گرم ماست معمولی به مدت 6 هفته دریافت نمودند. دریافت‌های غذایی، شاخص‌های تن سنجی و شاخص‌های بیوشیمیایی در ابتدا و انتهای مطالعه اندازه گیری شدند. تحلیل آماری با استفاده از نرم‌افزار SPSS و آزمون‌های تی زوجی و تحلیل کوواریانس انجام شد.
